# Reply to: “On the understanding of current-induced spin polarization of three-dimensional topological insulators”

**DOI:** 10.1038/s41467-019-10396-6

**Published:** 2019-06-07

**Authors:** C. H. Li, O. M. J. van ‘t Erve, S. Rajput, L. Li, B. T. Jonker

**Affiliations:** 1 Materials Science and Technology Division, Naval Research Laboratory, Washington, DC 20375 USA; 2 Intel Corporation, Hillsboro, OR 97124 USA; 3 Department of Physics and Astronomy, West Virginia University, Morgantown, WV 26506 USA

**Keywords:** Magnetic properties and materials, Spintronics, Topological insulators

## Introduction

**Replying To** J. Tian et al. *Nature Communications* 10.1038/s41467-019-09271-1 (2019)

Spin potentiometric measurements have been used to electrically detect spin polarization arising from spin-momentum locking in topological insulator (TI) surface states^1–10^, albeit with conflicting signs of the measured spin voltage. A model based upon spin-dependent potentials in the TI channel^4^ has often been used to interpret these data. We used this model in ref. ^9^, but have subsequently concluded that it is misleading and does not consider the real and more complex situation of the actual measurement.

This model postulates parallel spin-dependent potentials in the TI channel, and the issue raised by Tian et al.^11^ is the specific ordering of the spin-up and spin-down electrochemical potentials^4^. We had placed the spin-up (majority) electrochemical potential level further from zero due to its greater magnitude^9^, which may be inconsistent with their original model.

We subsequently realized that this model neglects critical spin-dependent parameters such as interface and channel resistances, and believe that is too simplistic to accurately account for the real experimental systems under study. Specifically, the potential profiles include vertical drops at the contact interface to the TI channel, which would indicate a current discontinuity (i.e., an infinite current), and therefore is fundamentally unphysical. It is this inadequacy which has resulted in apparent contradictory results from different groups. We have developed a more realistic model that includes experimental parameters such as interface and spin-dependent channel resistances, and find that a crossing of the spin-up and spin-down voltage levels can occur, which can lead to measured spin voltages of either sign^12^.

**Fig. 1:**
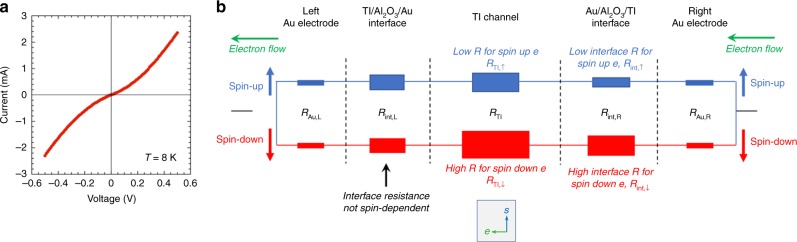
Typical I-V and schematic of model taking into account spin-dependent interface resistances. **a** Typical I-V curve taken at 8K between current injecting Au/Al_2_O_3_/Bi_2_Se_3_ contacts, showing a nonlinear behavior. **b** Schematic of a resistor circuit model for spin-up and spin-down electrons traveling in two independent channels from the right to the left electrode, where each component of the circuit from the contacts to interfaces are modeled as a resistor, specifically, the right Au electrode *R*_Au,R_, the right Au/Al_2_O_3_/TI interface *R*_int,R_, TI channel resistance *R*_TI_, the left TI/Al_2_O_3_/Au interface *R*_int,L_, and the left Au electrode *R*_Au,L_

Any practical system will have contacts for experimental access, and these interfaces are crucial to electrical transport when current is converted from one type of charge/spin carrier to another. The interface resistances at the current injecting contacts may be nonlinear and/or comparable to the TI channel, perhaps due to oxidation or a blanket layer of tunnel barrier material such as Al_2_O_3_ often deposited for capping purposes and/or to simplify fabrication^1,2,4–10^. This creates varying interface resistances that can fundamentally change the carrier conversion at the interface. Figure 1a shows a typical *I-V* curve taken between the current injecting Au/Al_2_O_3_/Bi_2_Se_3_ contacts as used in^1,9,12^, showing a non-negligible and nonlinear interface resistance where a voltage drop can be supported.

Figure 1b shows a schematic resistor circuit model for spin-up and spin-down electrons traveling in two independent channels^12,13^. As electrons travel from the right gold electrode to the left, different resistances are encountered which can be either spin independent, e.g., at the left Au/Al_2_O_3_/TI interface where electrons enter into the Au electrode (*R*_int,R↑_ = *R*_int,R↓_); or spin-dependent, e.g., the interface resistance where the electrons enter the TI channel (depending on the alignment of the electron spin and the states in the TI^14^ (*R*_int,R↑_ < *R*_int,R↓_). A detailed discussion is presented in^12^.

**Fig. 2:**
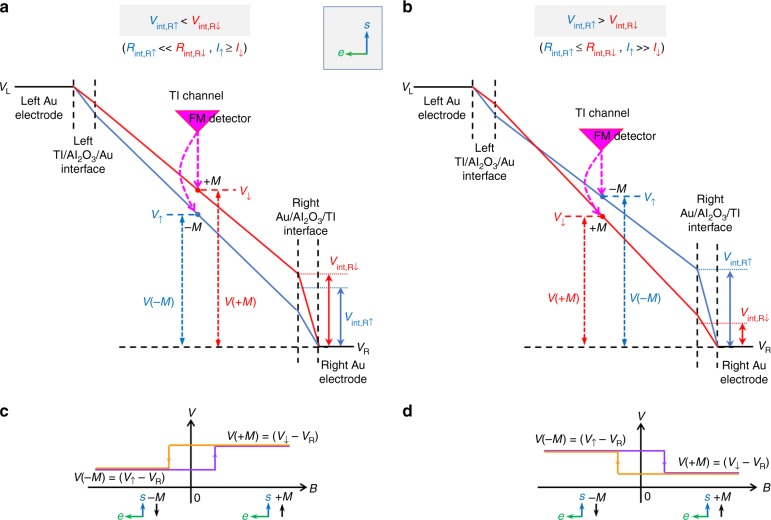
Voltage profile and predicted lineshape of measured spin voltages. Voltage profiles for the spin-up (blue) and spin-down (red) electrons for a left flowing current, for the case (**a**) *V*_int,R↑_ < V_int,R↓_ (due to for example *I*_*↑*_ ≥ I_↓_, *R*_int,R↑_ « *R*_int,R↓_), and (**b**) *V*_int,R↑_ > *V*_int,R↓_ (due to for example *I*_↑_ » *I*_↓_, *R*_int,R↑_ *≤* *R*_int,R↓_). Predicted lineshape for the spin voltage measured by a ferromagnet/tunnel barrier detector contact for the voltage profiles in (**a**, **b**) are shown in (**c**, **d**), respectively

Given that the overall voltage drop between the Au contacts must be the same for the spin-up and down channels, and that the spin-up channel is lower resistance, more current flows through the spin-up channel, *I*_↑_ > *I*_↓_. Consequently, at the left TI/Al_2_O_3_/Au interface that is spin *in*dependent, the voltage drop is greater for the spin-up channel, as depicted by the steeper slope for the spin-up (blue) line within the left interface region in Fig. 2a, b.

Conversely, at the right Au/Al_2_O_3_/TI interface that is spin-dependent, two different outcomes are possible depending upon the relative magnitudes of *R*_int,R↑_ and *R*_int,R↓_: either *V*_int,R↑_ < *V*_int,R↓_ or *V*_int,R↑_ > *V*_int,R↓_. This leads to potential profiles either with the spin-down level consistently above the spin-up level (Fig. 2a), or a crossing of these levels at some point within the TI channel (Fig. 2b).

It is important to recognize that this crossing does not imply a change in sign of the spin polarization in the channel or direction of the charge flow, but will result in a reversal of the measured spin voltage loop, as discussed below. Here charge carrier conversion at the Au/TI interfaces creates boundary conditions to ensure the spin and current continuity across the interface, just like carrier conversion at any ferromagnet/nonmagnet interfaces^12,13^. This results in the splitting of the spin-up and spin-down levels near the interface in the TI. However, the spin coherence length in the TI surface states is very large and typically greater than the TI channel length. Hence the equilibrium conditions that would be expected for an isolated TI are not reached. Thus different voltage profiles and relative energies of the spin-up and spin-down levels can be created depending on the relative magnitudes of the spin-dependent resistances at interfaces and within the channel. A detailed treatment is provided in ref. ^12^.

Probing these voltage profiles by a ferromagnet/tunnel barrier contact via potentiometric measurement^9^ yields hysteresis loops of opposite sign as shown in Fig. 2c, d. The sign in Fig. 2c is consistent with observations in refs. ^4,6,8^, and that of Fig. 2d consistent with refs. ^1,8–10^.

As noted above, the resistances at the left TI/Al_2_O_3_/Au and right Au/Al_2_O_3_/TI interfaces are not symmetric, and can be non-ohmic and/or rectifying, due to TI surface oxidation or inclusion of a tunnel barrier^1,2,4–10^. This can lead to a larger splitting between the spin-up and spin-down voltage levels at the higher resistance interface (right contact), pushing the crossing towards the opposite end of the TI channel (Fig. 2b)^12^.

Regarding the work on current generated spin in InAs^11^, we note that different physical quantities were measured: Johnson/Hammar presented their results in voltage (Refs. 9,10. in ref. ^11^), whereas Park et al. reported resistance (Refs. 11,12. in ref. ^11^, which cannot be compared for sign. These papers also lacked adequate documentation of current, magnetic field direction, and/or polarities of voltage probes to enable one to establish a sign convention for their reported spin polarization, and used different quantum well structures. However, they all report a positive value for the Rashba spin-orbit coupling coefficient, α, which is more fundamental and directly addresses the spin ordering of the states. We specifically addressed this in ref. ^9^.

In summary, the original model^4^ adapted in ref. ^9^ is too simplistic and unphysical, and any discussion of the precise placement of the spin potentials is irrelevant to the determination of the sign and origin of the measured spin voltage. Our revised model remedies this by taking into account common experimental parameters such as spin-dependent channel and interface resistances, which can have a critical impact on the resultant spin voltage measured. Our conclusion^9^ that the spin signal we measured originates from Bi_2_Se_3_ Dirac surface states is sound. It is independently supported by the different temperature dependences observed for Bi_2_Se_3_ and InAs, and the self-cancellation of contributions from the spin-split 2DEG states which potentially coexist on the Bi_2_Se_3_ surface^15^. The opposite sign of the measured spin voltage we reported for Bi_2_Se_3_ relative to the InAs reference provides corroborative evidence, but we now realize is insufficient on its own unless the analysis includes the details of our new model^12^. The InAs(001) surface 2DEG provides an excellent reference for the sign of the spin voltage, because no additional states are present, the orientation of the effective electric field is well established (no modulation doping), and ultra-low resistance ohmic contacts can be obtained. Finally, our findings potentially reconcile the inconsistencies reported in the literature, and underscore the importance of recognizing these contributions in the interpretation of such data.

## Data Availability

The data sets generated during and/or analyzed during the current study are available from the corresponding author on reasonable request.
